# Long‐Term Survival, Burden of Disease, and Patient‐Centered Outcomes in Maximally Treated Intracerebral Hemorrhage

**DOI:** 10.1002/acn3.70048

**Published:** 2025-04-14

**Authors:** Anne Mrochen, Maximilian I. Sprügel, Alexander Sekita, Stefanie Balk, David Haupenthal, Stefan T. Gerner, Hannes Lücking, Arnd Doerfler, Kosmas Macha, Stefan Schwab, Joji B. Kuramatsu, Jochen A. Sembill

**Affiliations:** ^1^ Department of Neurology University Hospital Erlangen Erlangen Germany; ^2^ Department of Neuroradiology University Hospital Erlangen Erlangen Germany

**Keywords:** intracerebral hemorrhage prognosis, patient‐centered outcomes, stroke disability burden

## Abstract

**Objective:**

Increasing evidence shows that patients with intracerebral hemorrhage (ICH) can achieve better‐than‐expected outcomes with aggressive therapy. However, real‐world long‐term data, patient‐centered outcomes, and societal measures after maximal ICH treatment are lacking. This study aimed to analyze 5‐year survival, utility‐weighted functional outcomes, and burden of disease in maximally treated ICH patients, stratified by max‐ICH Score.

**Methods:**

This study investigated consecutive patients with spontaneous ICH included in the single‐center Longitudinal Cohort Study on ICH Care (UKER‐ICH, NCT03183167, 2006–2015). We included all patients without early care limitations, hereinafter referred to as maximally treated. We analyzed the stratification by max‐ICH Score of cumulative 5‐year survival using Kaplan–Meier estimates and COX regression modeling, disease burden using disability‐adjusted life years (DALYs), and patient‐centered outcome at 12 months using the Utility‐Weighted modified Rankin Scale (UW‐mRS).

**Results:**

The 5‐year survival rate of the included 1022 maximally treated patients was 53%, stratified by max‐ICH Score (0 points: 85%, 1: 91%, 2: 69%, 3: 59%, 4: 47%, 5: 32%, 6: 29%, 7: 18%, ≥ 8: 0%, log‐rank *p* < 0.001). The mean number of DALYs was 8.94 (±8.15, standard deviation [SD]), consisting of 4.27 years of life lost (±7.79, SD) and 4.67 years lived with disability (±6.38, SD). Patients with a max‐ICH Score of 5 had the highest burden of disease (12.76 [±9.43, SD]). The mean UW‐mRS at 12 months was 0.45 (±0.37, SD) and decreased with increasing max‐ICH Score (0: 0.80 [±0.23], 1: 0.73 [±0.29], 2: 0.67 [±0.29], 3: 0.50 [±0.34], 4: 0.39 [±0.34], 5: 0.25 [±0.30], 6: 0.19 [±0.28], 7: 0.16 [±0.26], ≥ 8: 0.08 [±0.22], *p* < 0.001).

**Interpretation:**

These observational data, stratified by max‐ICH Score, provide patients and treating physicians with an initial severity assessment in terms of potential long‐term patient‐centered outcomes and burden of disease following maximal treatment.

## 1 Introduction

In recent years, there has been increasing evidence that patients with intracerebral hemorrhage (ICH) can achieve a favorable outcome following an aggressive therapeutic approach [1]. Numerous studies have shown that care limitations in the form of withholding or withdrawing life‐sustaining therapies are independently associated with an increased risk of mortality and unfavorable functional outcomes when initiated early (usually within the first day) after ICH [1, 2, 3]. The avoidance of early care limitation revealed that this self‐fulfilling prophecy of poor outcomes could be averted [4]. Patients with severe ICH who have participated in randomized trials have demonstrated functional long‐term outcomes after aggressive treatment that exceeded expectations, irrespective of the nonsignificant results of the interventions studied [5]. Furthermore, the implementation of bundle care treatment for ICH has led to improved outcomes, as has the use of minimally invasive and decompressive surgical options [6, 7, 8]. However, there is a lack of real‐world long‐term data focusing on patient‐centered outcomes and the burden of living with disability for an individual's remaining life after maximal treatment, as well as a methodology to stratify each patient's probability in this regard [1, 9]. The max‐ICH Score is widely recognized as a prognostic tool for ICH with minimized confounding by false‐positive unfavorable outcome attribution. It has been validated across multiple centers and settings and has demonstrated its clinical utility in predicting mortality and functional outcomes up to 12 months [1, 10, 11, 12]. The objective of the present study was to analyze, for the first time, the 5‐year overall survival, utility‐weighted functional outcomes, and burden of disease in patients with ICH after maximal therapy, and to analyze their stratification by the max‐ICH Score.

## 2 Methods

### 2.1 Study Design and Participants

The present study investigated patients with spontaneous ICH included in the Longitudinal Cohort Study on ICH Care (UKER‐ICH, NCT03183167) of the University Hospital Erlangen. The detailed methodology has been previously published [10, 13, 14]. In summary, this prospective, single‐center cohort study included 1322 consecutive patients with spontaneous ICH admitted to the University Hospital Erlangen between January 1, 2006, and December 31, 2015. Patients with secondary ICH because of aneurysm, intratumoral hemorrhage, trauma, arteriovenous malformation, thrombocytopenia < 50,000/mL, or following thrombolysis were excluded a priori [10, 13, 14]. As previously described, we further excluded patients with missing data for max‐ICH Score calculation, aggressiveness of care or follow‐up (*n* = 115), and patients with early care limitations (*n* = 185) (see Figure S1 for the patient flow diagram) [10, 11]. Early care limitations were defined as withholding or withdrawal of potentially life‐sustaining therapy within the first 24 h after admission [10, 11]. This included mechanical ventilation, cardiopulmonary resuscitation, the use of vasopressors, antibiotics, or intracranial surgery (such as the placement of external ventricular drain or hematoma evacuation) [10]. In the absence of other forms of limitation, do‐not‐resuscitate (DNR) orders did not constitute a limitation of care [10, 11]. These care limitations, if initiated early, could potentially lead to a self‐fulfilling prophecy, as the outcome after aggressive therapy might be more favorable than expected [10, 11]. The study population consisted of 1022 patients treated at a Western university hospital with a dedicated neurointensive care unit. These patients were not subjected to any early care limitations and received full intensive care treatment in accordance with established guidelines, and thus were considered to have received maximum treatment [10].

### 2.2 Data Acquisition

Baseline data including demographic information, medical history, neurological status, neuroradiological data, and in‐hospital parameters were obtained as reported earlier [10, 13, 14, 15]. The max‐ICH Score was assessed for each patient, considering age, National Institutes of Health Stroke Scale (NIHSS) score, intraventricular hemorrhage, intake of anticoagulation, and ICH volume (lobar and nonlobar), see Table S1 [10, 11]. Follow‐up data were assessed by standardized mailed questionnaires or semistructured telephone interviews at 3 and 12 months, and thereafter retrieved from institutional databases in case of hospital readmission, as previously described [14, 16]. Functional outcomes were evaluated using the modified Rankin Scale (mRS) categorized as favorable (mRS 0–3) or unfavorable (mRS 4–6). We calculated patient‐centered utility‐weighted mRS (UW‐mRS) scores by applying weighting factors identified in previous studies [17]. Utility weights were employed to convert the spacing between the seven ranks on the mRS from arbitrarily uniform intervals to distances that accurately reflect the patient and societal valuation of each outcome state [18, 19]. The burden of disease was assessed for each patient by calculation of disability‐adjusted life years (DALYs) using follow‐up data on functional outcome at 12 months and survival time, as previously described [15]. It represents the sum of years of life lost (YLL) due to premature mortality, that is, the difference between the patient's age‐specific life expectancy and age at death, and years lived with disability (YLD), that is, the number of YLD multiplied by a disability‐weighting factor, following the established classification by the World Health Organization [15, 20]. Accordingly, the calculation did not include premorbid status, age weighting, or future discount [15, 20]. YLDs were calculated for three time periods: from hospital discharge to 3 months after ICH diagnosis, from 3 to 12 months after ICH, and from 12 months after ICH to death [15]. The most recent functional status was applied for each time interval [15]. The life expectancy of the general population was obtained from mortality tables specific to age and sex, published by the German Federal Statistical Office [21]. In essence, the DALY represents a time‐based measure of health status that incorporates both disability and mortality [9]. One DALY is thus equivalent to one year lost of healthy life [9].

### 2.3 Statistical Analysis

Statistical analyses were performed with SPSS version 28.0.0.0 (SPSS Inc., Chicago, IL) and Stata version 18. We considered a 2‐tailed *p* < 0.05 to be statistically significant. Normally distributed data are presented as mean (±standard deviation [SD]); non‐normally distributed data as median and interquartile range (IQR). We analyzed the cumulative survival estimates over 5 years using Kaplan–Meier estimates and compared the stratification by the max‐ICH Score applying the log‐rank test. Patients who were lost to follow‐up were censored at their last known follow‐up date, whereas deaths were included as events in the analysis. We used Cox regression modeling to calculate the hazard ratio (HR) with corresponding 95% confidence interval (CI), which assesses the hazard of death associated with different max‐ICH Scores relative to a max‐ICH Score of 0 (reference). Analysis of variance was employed to conduct intergroup comparisons based on patients' max‐ICH Scores, analyzing the burden of disease as measured by DALYs and patient‐centered functional outcome at 12 months as measured by UW‐mRS.

### 2.4 Standard Protocol Approvals, Registrations, and Patient Consents

The UKER‐ICH study was approved by the local institutional review board, and informed consent was obtained from patients or their legal representatives.

## 3 Results

### 3.1 Patient Characteristics

We investigated 1022 maximally treated patients with spontaneous ICH. The mean age of the study population was 69.5 (±12.9) years, and the median hematoma volume was 13.0 mL (IQR 4.9–30.5) with intraventricular extension in 394 (38.5%) patients, resulting in overall moderate neurological impairment (median NIHSS 9, IQR 4–18), see Table 1 for patient characteristics. Patients were classified according to the severity of ICH, as determined by the max‐ICH Score (median 4, IQR 2–5). The median length of hospital stay was 11 (IQR 7–17) days. 277 (27.1%) patients required external ventricular drainage, and 73 (7.1%) required hematoma evacuation. The number of patients who died during hospitalization was 66 (5.2%), and those who survived were discharged with a median modified Rankin Scale score of 4 (IQR 3–5).

**TABLE 1:** Characteristics of maximally treated patients with intracerebral hemorrhage.

Characteristics	Patients (*n* = 1022)
Age, years, mean (SD)	69.5 (12.9)
Sex, female, *n* (%)	462 (45.2%)
Medical history	
Hypertension, *n* (%)	877 (85.8%)
Diabetes mellitus, *n* (%)	281 (27.5%)
Oral anticoagulation, *n* (%)	158 (15.5%)
Antiplatelet use, *n* (%)	307 (30.0%)
Neurological status	
GCS, median (IQR)	14 (11–15)
NIHSS, median (IQR)	9 (4–18)
Imaging	
Hematoma volume, mL, median (IQR)	13.0 (4.9–30.5)
Lobar ICH location, *n* (%)	441 (43.2%)
Nonlobar ICH location, *n* (%)	581 (56.8%)
Intraventricular hemorrhage, *n* (%)	394 (38.5%)
In‐hospital data	
External ventricular drain, *n* (%)	277 (27.1%)
Length of hospital stay, days, median (IQR)	11 (7–17)
Hematoma evacuation, *n* (%)	73 (7.1%)
mRS 6 at discharge, *n* (%)	66 (6.5%)
mRS at discharge, median (IQR)	4 (3–5)

Abbreviations: GCS, Glasgow Coma Scale (ranging from 3, comatose, to 15, alert); ICH, intracerebral hemorrhage; IQR, interquartile range (25th to 75th percentile); mRS, modified Rankin Scale (0 no deficit to 6 death); NIHSS, National Institutes of Health Stroke Scale (ranging from 0, no deficit, −40, severe neurological deficit; 40 is the maximum because in comatose ataxia is not scored); SD, standard deviation.

### 3.2 Five‐Year Survival

**FIGURE 1:**
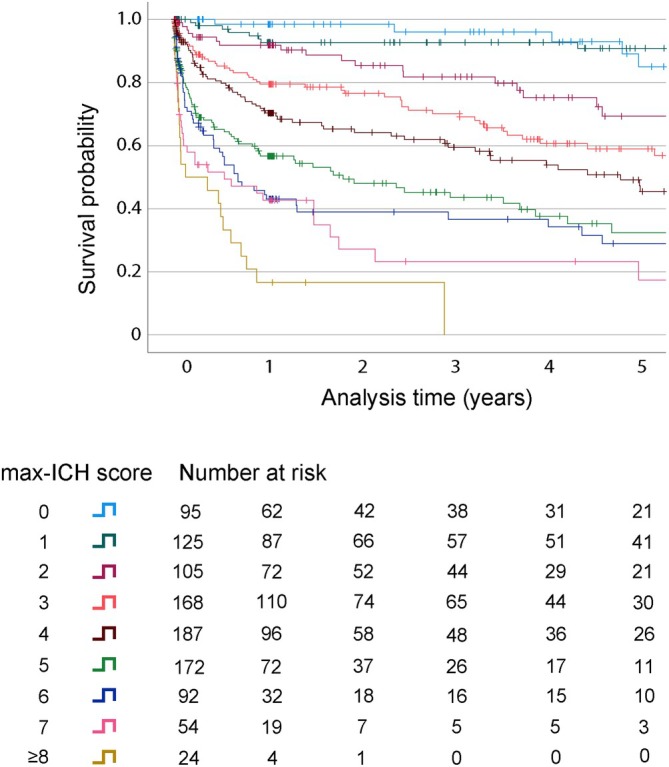
Kaplan–Meier survival curves after maximal treatment stratified by the max‐ICH Score. Kaplan–Meier survival curves illustrate the survival probabilities over a 5‐year period. The curves are segmented according to max‐ICH Scores, highlighting differences in survival outcomes based on the severity of the initial ICH. The “Number at Risk Table” provides the count of patients at the beginning of each annual interval for each max‐ICH Score group.

**FIGURE 2:**
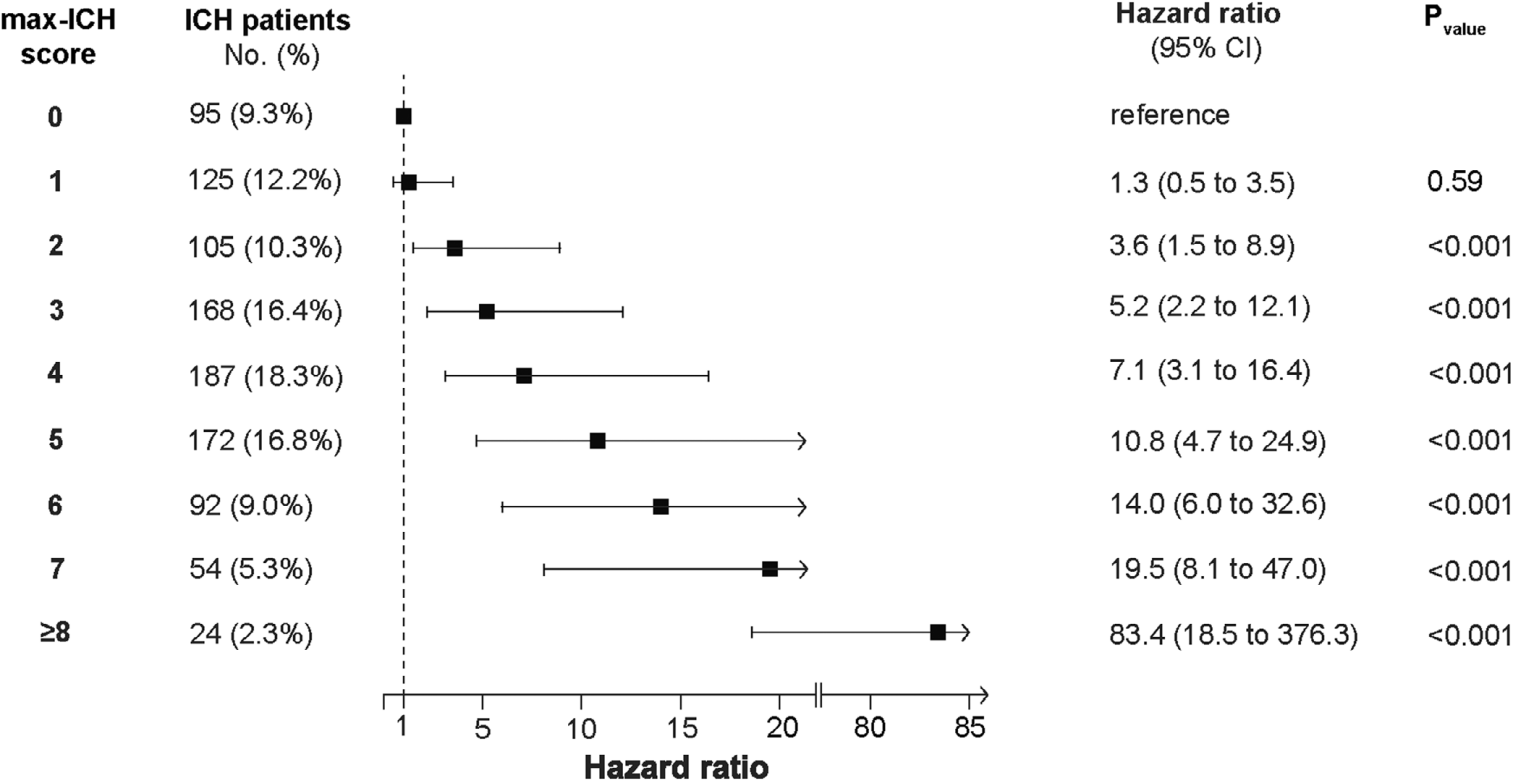
Hazard of mortality after maximal treatment stratified by the max‐ICH Score. Cox proportional hazards regression analysis was conducted to assess the relative hazard of death, with 95% confidence intervals (CI), associated with each max‐ICH Score. The reference group for this analysis was the group with a max‐ICH Score of 0. The results illustrate the relationship between increasing max‐ICH Scores and the hazard of death. Abbreviations: CI, confidence interval; ICH, intracerebral hemorrhage.

Kaplan–Meier analysis was performed to assess the survival of maximally treated patients over a 5‐year period after ICH. The median follow‐up time was 1.03 years (IQR [years] 0.10–3.62). The 1‐, 2‐, 3‐, 4‐, and 5‐year survival estimates for the total cohort were 71%, 67%, 63%, 59%, and 53%, respectively. We calculated Kaplan–Meier survival curves for each max‐ICH Score, as illustrated in Figure 1. The max‐ICH Score stratified patient's probability for survival over 5 years (log‐rank *p* < 0.001). The corresponding 5‐year survival estimates for each max‐ICH Score were as follows (max‐ICH Score: survival rate): 0: 85%, 1: 91%, 2: 69%, 3: 59%, 4: 47%, 5: 32%, 6: 29%, 7: 18%, and ≥ 8: 0%. We conducted Cox proportional hazards regression analysis to assess the relative hazard of death associated with each max‐ICH Score, with max‐ICH Score 0 serving as a reference, see Figure 2. This analysis showed an increasing hazard ratio (HR) for mortality with increasing max‐ICH Scores, with significant results from the comparison of a max‐ICH Score of 2 or higher compared to 0.

### 3.3 Burden of Disease

**FIGURE 3:**
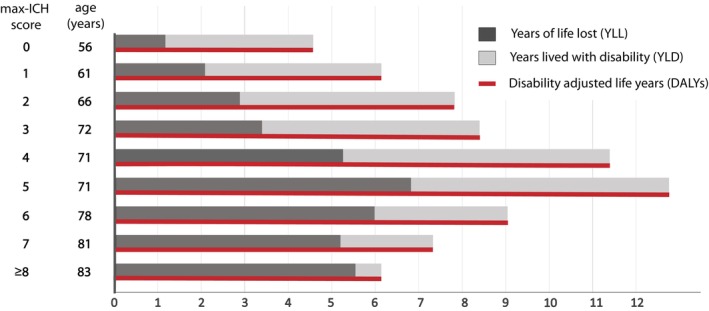
The burden of disease after maximal treatment stratified by the max‐ICH Score. The number and subdivision of disability‐adjusted life years (DALYs) were analyzed for each max‐ICH Score. The red bar represents the total DALYs, which are composed of years lived with disability (YLD, light gray bar) and years of life lost (YLL, dark gray bar). To assist in the interpretation of the combined analysis of morbidity and mortality, the average age (in years) of the patients with individual max‐ICH Scores is also provided.

We analyzed the burden of disease measured by DALYs of maximally treated patients after ICH using the observed functional outcome at 12 months and survival data. The observed mean number of DALY after ICH was 8.94 (±8.15), consisting of 4.67 YLD (±6.37) and 4.23 YLL (±7.79). The number and subdivision of DALYs were analyzed for each max‐ICH Score, as shown in Figure 3. The corresponding DALYs for each max‐ICH Score were as follows (max‐ICH Score: DALY [±SD]): 0: 4.58 [±5.65], 1: 6.14 [±7.70], 2: 7.83 [±6.91], 3: 8.40 [±6.94], 4: 11.39 [±9.86], 5: 12.76 [±9.43], 6: 9.05 [±6.55], 7: 7.35 [±3.35], and ≥ 8: 6.45 [±2.08], *p* < 0.001 for intergroup comparison. The burden of disease by max‐ICH Score therefore increased to a maximum value of 12.76 DALYs at 5 points, and then decreased again with increasing age at disease onset.

### 3.4 Patient‐Centered Long‐Term Outcome at 12 Months

**FIGURE 4:**
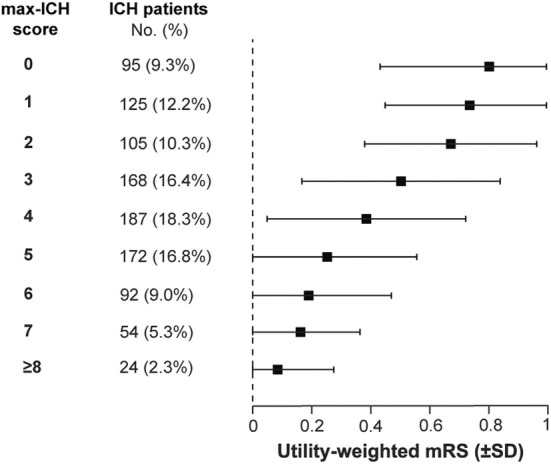
Patient‐centered functional long‐term outcome after maximal treatment stratified by the max‐ICH Score. The patient‐centered functional outcome 12 months after maximal treatment was analyzed using the utility‐weighted mRS. Results are presented separately for each max‐ICH Score, indicating the mean UW‐mRS and standard deviation (SD). Abbreviations: ICH, intracerebral hemorrhage; mRS, modified Rankin Scale.

We analyzed the patient‐centered functional outcome 12 months after maximal treatment using the UW‐mRS. The mean UW‐mRS at 12 months for the entire cohort was 0.45 (±0.37 SD). The mean UW‐mRS decreased with increasing max‐ICH Score (max‐ICH Score: UW‐mRS [±SD]): 0: 0.80 [±0.23], 1: 0.73 [±0.29], 2: 0.67 [±0.29], 3: 0.50 [±0.34], 4: 0.39 [±0.34], 5: 0.25 [±0.30], 6: 0.19 [±0.28], 7: 0.16 [±0.26], and ≥ 8: 0.08 [±0.22], documenting valid stratification of patient‐centered outcomes by max‐ICH Score (*p* < 0.001 for intergroup comparison); see Figure 4.

## 4 Discussion

This single‐center cohort study of 1022 patients with maximally treated ICH provides real‐world data on 5‐year survival and utility‐weighted functional outcome, stratified by the max‐ICH Score. A combined time‐based analysis of mortality and morbidity by DALYs yielded a quantification of the patient and societal burden following maximally treated ICH.

The cumulative 5‐year survival rate of 53% is in the upper range of previous studies (27%–57%), which might indicate a trend toward improved survival estimates following maximal therapy [22, 23, 24, 25]. However, direct comparisons are limited by differences in demographic characteristics between the study populations [22, 23, 24, 25]. Our findings documented an association between higher max‐ICH Scores and lower 5‐year survival estimates, emphasizing the prognostic importance of this score for long‐term outcomes beyond 12 months. It can be hypothesized that the stratification of long‐term mortality by the max‐ICH Score is attributable to the integration of patient age, a potential association of cardiovascular risk, and the use of antithrombotic medication with ICH severity, as well as the reduced life expectancy due to remaining disability after ICH [26, 27]. The only categories of max‐ICH Scores in which no significant difference in mortality hazard was observed were scores 0 and 1. However, the survival estimates observed in patients with scores ranging from 0 to 2 (69%–85%) were even comparable to those observed in a control group of similar age without ICH [25]. This suggests that long‐term survival after maximal therapy is not diminished in younger patients with mild ICH. Furthermore, it indicates the potential efficacy of aggressive treatment strategies to meaningfully improve patient outcomes, highlighting the need for further research and refinement of treatment bundles and surgical options, as recently demonstrated [6, 7, 8].

The increasing recognition of the importance of comprehensive and patient‐centered outcome assessment has led to the increasing use of the health utility‐weighted mRS, which converts the spacing between the seven ranks on the mRS from arbitrarily uniform intervals to distances that accurately reflect the patient and societal valuation of each outcome state [18, 28]. Our data show that the max‐ICH Score also stratifies this utility‐weighted outcome and thus provides a patient‐centered severity assessment for patients, relatives, and practitioners to support the planning of treatment and recovery goals after maximal therapy.

With regard to the disease burden, there is a discrepancy between the continuous increase in the maximum level of the mRS or UW‐mRS score and the course of the assigned DALYs. The DALYs did not reach their highest point at the max‐ICH Score of 9, as would be anticipated, but rather at score 5, followed by score 4. This pattern indicates that patients with mid‐range max‐ICH Scores experience the greatest burden of disease. Given that the max‐ICH Score incorporates patient age into its severity assessment, patients with a very high score are also older and, as a consequence, have a statistically lower remaining life expectancy at disease onset. This, in turn, translates into lower YLL and YLD. As illustrated in Figure 3, patients with a max‐ICH Score of 0–2 are, on average, below the age of 70, whereas those with a score of 3–6 are below the age of 80, and those with a score of 7–9 are above the age of 80. Consequently, the observation that the disease burden is decreasing again should not be interpreted as an indication of a lower severity of the individual ICH. Rather, it must be interpreted in the context of a societal measure that is influenced by statistical life expectancy. Overall, we report a substantial health burden following the maximal treatment of ICH, with an average of 8.94 (±8.15) DALYs. For context, we note that this figure is considerably greater than reported for severe ischemic stroke (5.9 DALYs) [29]. Observed DALYs here were slightly below the reported number of DALYs from a full‐cohort analysis of the UKER‐ICH registry (9.46 ± 8.08), which also included patients with care limitations [15]. Furthermore, we observed a higher fraction of YLD (4.67 ± 6.37) compared to YLL (4.23 ± 7.79) after maximal treatment compared to the full‐cohort analysis (YLD: 3.74 ± 5.95) and YLL (5.72 ± 8.29) [15]. Accordingly, a priori excluded patients with early care limitations within the first 24 h had a mean DALY of 11.3, driven exclusively by YLL (data not shown). We therefore hypothesize that with potentially improved survival rates after aggressive treatment, disability becomes a larger component of the disease burden and healthcare expenditures [20]. This also calls for greater research efforts to identify new, more effective intervention strategies to improve functional outcomes after ICH.

It is important to note that certain limitations exist with regard to the interpretation of our results. First, the study was monocentric, which may limit the generalizability of the results to other centers. Additionally, given the long‐term outcome assessment, it is inevitable to acknowledge changes in the intensity and management strategies of ICH care over the past decade. Second, the study represents a retrospective analysis of observational data, which carries the risk of relevant confounding, particularly due to selection bias regarding maximally treated patients, as previously described [10]. Nevertheless, it is unlikely that randomized studies will be conducted in this context due to ethical considerations [1]. Analyzing the outcomes of patients without early care limitations may further result in a severity bias, as these patients could be expected to have an overall better prognosis regardless of individual severity assessed using a prognostic model [11, 30]. Furthermore, DNR orders, in isolation, not designated as a care limitation, may have exerted an unmeasured influence on the aggressiveness of care and the adoption of surgical procedures in clinical practice. The present approach resulted in a cohort that is comparable in main clinical characteristics to other large observational studies and clinical trials [31]. However, the application of prognostic information derived from patient cohorts to individual patients is inevitably associated with a certain degree of uncertainty and should therefore not be regarded as the sole factor in predicting outcome [1, 11]. Third, utility weighting varies based on the cohort and the choice of health utility scale [28]. We applied conversions that had not been validated for outcome assessment after maximally treated ICH [17]. However, we believe that these conversions are suitable for the purpose of validating the stratification of the patient‐centered severity of ICH. The aim of making an individual patient‐centered outcome prediction was not pursued because of the aforementioned limitations. Fourth, the analysis does not include information on the impact of preexisting conditions, posttreatment events, or subsequent aggressiveness of care on outcome measures. In light of these variables, it is reasonable to hypothesize that additional potential confounders may have exerted an influence on the subsequent course of disease in this real‐world observational cohort of consecutive patients with ICH as a qualifying event. Additionally, the analysis did not take hematoma location into account. Although the distinction between lobar and nonlobar locations is inherently integrated into the score calculation, the nuanced impact of anatomical location was not captured. Finally, despite the overall large number of patients included, the smaller number within individual max‐ICH categories, especially those with high point values, may have resulted in a reduction of prognostic precision.

This study presents, for the first time, real‐world data on 5‐year survival and 12‐month utility‐weighted functional outcome of patients with ICH who have undergone maximal treatment, as well as their valid stratification by the max‐ICH Score. The incorporation of societal measures into the analysis provides a quantitative assessment of the impact of maximally treated ICH. These data further validate the max‐ICH Score as part of the multimodal assessment of ICH patients and may assist in the evaluation of long‐term treatment and recovery goals, as well as the development of public health strategies.

## Author Contributions

Conceived and designed the analysis: J.A.S. Collected the data: J.A.S., M.I.S., D.H., J.B.K. Contributed data or analysis tools: H.L., A.D. Performed the analysis: A.M., D.H., J.A.S. Wrote the paper: A.M., J.A.S. Critically revised the manuscript: M.I.S., A.S., S.B., D.H., S.T.G., H.L., A.D., K.M., S.S., J.B.K.

## Disclosure

M.I.S. reported receiving grants from the Interdisziplinäres Zentrum für Klinische Forschung, Marohn Foundation, Doktor Robert Pfleger‐Stiftung, and German Society for Neurointensive Care and Emergency Medicine (DGNI) for work outside the submitted work.

## Conflicts of Interest

A.M. reports personal fees from Alexion Pharma Germany GmbH and Roche Pharma GmbH, outside the submitted work. J.A.S. reports personal fees from Lilly Deutschland GmbH, outside the submitted work.

## Supporting information


Data S1

## Data Availability

The data that support the findings of this study are available from the corresponding author, J.A.S, upon reasonable request.
