# Supplementary material for: Long‐Term Survival, Burden of Disease, and Patient‐Centered Outcomes in Maximally Treated Intracerebral Hemorrhage
Source: Ann Clin Transl Neurol. 2025 Apr 14;12(6):1144–50. doi: 10.1002/acn3.70048 (PMC12172108; doi:10.1002/acn3.70048)
Supplement: Supplementary file 1 — Data S1 [file ACN3-12-1144-s001.docx]

**Online Supplement**

Mrochen et al. *Five-Year Survival, Burden of Disease, and Patient-Centered Outcomes of*

*Patients with Intracerebral Hemorrhage following Maximal Treatment*

Supplemental Table 1. Definition of the max-ICH Score

Supplemental Figure 1. Flow diagram of study participants

**Supplemental Table 1. Definition of the max-ICH Score** ^1^

| ***Component*** | ***Points*** |
| --- | --- |
| NIHSS | |
| 0-6 | 0 |
| 7-13 | 1 |
| 14-20 | 2 |
| ≥21 | 3 |
| Age, y | |
| ≤69 | 0 |
| 70-74 | 1 |
| 75-79 | 2 |
| ≥ 80 | 3 |
| Intraventricular hemorrhage | |
| No | 0 |
| Yes | 1 |
| Oral anticoagulation | |
| No | 0 |
| Yes | 1 |
| Lobar ICH volume, mL | |
| <30 | 0 |
| ≥30 | 1 |
| Nonlobar ICH volume, mL | |
| <10 | 0 |
| ≥10 | 1 |
| Total max-ICH Score | 0 - 10 |

The score must be determined on the basis of initial imaging using CT or MRI. Lobar ICH refers to an ICH that originates at the cortex and cortical-subcortical junction. Nonlobar ICH includes hemorrhage originating from deep brain structures, the cerebellum, or the brainstem. Deep ICH is specifically defined as hemorrhage involving the basal ganglia, thalamus, internal capsule, and deep periventricular white matter. If an ICH spans both deep and lobar locations, it should be scored based on the location where the hemorrhage most likely originated. Consequently, obtaining more than 1 point for ICH volume is only possible in the rare instance of having two distinct ICHs: one large lobar and one large nonlobar ICH.

Abbreviations: ICH, Intracerebral hemorrhage; NIHSS, NIH Stroke Scale; y, years.

Reference:

^1^ Sembill JA, Gerner ST, Volbers B, et al. Severity assessment in maximally treated ICH patients: The max-ICH score. Neurology. Aug 1 2017;89(5):423-431. doi:10.1212/wnl.0000000000004174

**Supplemental Figure 1.** Flow diagram of study participants


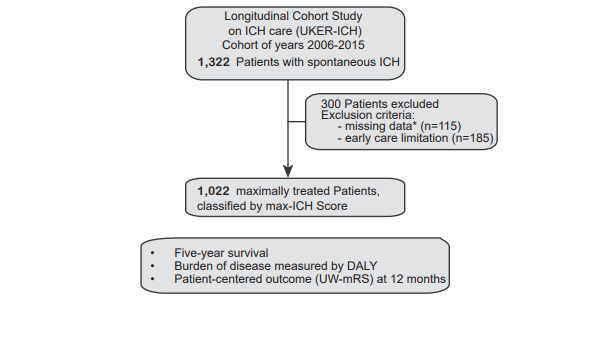


A total of 1,322 patients with spontaneous ICH (years 2006-2015) were screened for analysis. 300 patients were excluded because of missing data* for max-ICH Score calculation, aggressiveness of care or follow-up (n=115), and patients with early care limitations (n=185). 1,022 patients, classified according to max-ICH Score, were available for outcome analysis and were considered to have received the maximum treatment. The key outcomes assessed included 5-year survival, burden of disease measured by DALY (Disability-Adjusted Life Years) and patient-centered functional outcome at 12 months, evaluated using the utility-weighted mRS (UW-mRS).
